# Contact Laxative Use and the Risk of Arteriovenous Fistula Maturation Failure in Patients Undergoing Hemodialysis: A Multi-Center Cohort Study

**DOI:** 10.3390/ijerph19116842

**Published:** 2022-06-03

**Authors:** Trung Hoang Anh, Phung-Anh Nguyen, Anh Duong, I-Jen Chiu, Chu-Lin Chou, Yu-Chen Ko, Tzu-Hao Chang, Chih-Wei Huang, Mai-Szu Wu, Chia-Te Liao, Yung-Ho Hsu

**Affiliations:** 1 International Ph.D. Program in Medicine, College of Medicine, Taipei Medical University, Taipei 110, Taiwan; hoangtrung.doctor@gmail.com; 2 Nephro-Urology and Dialysis Center, Bach Mai Hospital, Ha Noi 100000, Vietnam; 3 Clinical Data Center, Office of Data Science, Taipei Medical University, Taipei 110, Taiwan; alexnthhp@gmail.com; 4 Clinical Big Data Research Center, Taipei Medical University Hospital, Taipei 110, Taiwan; 5 Department of Healthcare Information and Management, Ming Chuan University, Taoyuan 330, Taiwan; 6 Macquarie Business School, Macquarie University, Sydney, NSW 2109, Australia; ashley.duong2301@gmail.com; 7 Division of Nephrology, Department of Internal Medicine, Taipei Medical University-Shuang Ho Hospital, New Taipei City 235, Taiwan; stirbar2000@yahoo.com.tw (I.-J.C.); maiszuwu@gmail.com (M.-S.W.); 8 Division of Nephrology, Department of Internal Medicine, School of Medicine, College of Medicine, Taipei Medical University, Taipei 110, Taiwan; chulin.chou@gmail.com; 9 TMU-Research Center of Urology and Kidney (TMU-RCUK), Taipei Medical University, Taipei 110, Taiwan; 10 National Defense Medical Center, Division of Nephrology, Department of Medicine, Tri-Service General Hospital, Taipei 110, Taiwan; 11 Division of Nephrology, Department of Internal Medicine, Taipei Medical University-Hsin Kuo Min Hospital, Taoyuan City 330, Taiwan; 12 Division of Cardiovascular Surgery, Department of Surgery, Taipei Medical University-Shuang Ho Hospital, New Taipei City 235, Taiwan; koyuchen66@gmail.com; 13 Graduate Institute of Biomedical Informatics, College of Medical Science and Technology, Taipei Medical University, Taipei 110, Taiwan; kevinchang@tmu.edu.tw (T.-H.C.); gracehuang@tmu.edu.tw (C.-W.H.); 14 International Center for Health Information Technology, College of Medical Science and Technology, Taipei Medical University, Taipei 110, Taiwan

**Keywords:** contact laxatives, arteriovenous fistula, maturation failure, end-stage kidney disease, hemodialysis

## Abstract

Laxatives are commonly prescribed for constipation management; however, they are recognized as an independent factor associated with cardiovascular diseases. Arteriovenous fistula (AVF) is the closest to the ideal model of hemodialysis (HD) vascular access and part of the cardiovascular system. Our study aims to explore the association of contact laxative use with AVF maturation outcomes in patients undergoing HD. We conducted a multi-center cohort study of 480 contact laxative users and 472 non-users who had undergone initial AVF creation. All patients were followed until the outcomes of AVF maturation were confirmed. Multivariable logistic regression models were performed to evaluate the risk of AVF maturation failure imposed by laxatives. Here, we found that patients who used contact laxatives were significantly associated with an increased risk of AVF maturation failure compared to non-users (adjusted odds ratio, 1.64; *p* = 0.003). Notably, the risk of AVF maturation failure increased when increasing their average daily doses and cumulative treatment days. In conclusion, our study found a significant dose- and duration-dependent relationship between contact laxative use and an increased risk of AVF maturation failure. Thus, laxatives should be prescribed with caution in this population. Further studies are needed to validate these observations and investigate the potential mechanisms.

## 1. Introduction

Constipation is a common clinical problem that affects about 30% of the general population and is characterized by infrequent bowel movements, difficult stool passage, or both [1]. In hemodialysis (HD) patients, this condition is highly prevalent, with chronic or recurrent nature, and necessitates regular or frequent prescriptions of laxatives, partly because of their dietary/water restrictions, chronic medication use, high prevalence of comorbidities, lack of exercise, uremic toxins, and altered gut microbiota [2,3,4,5,6]. Based on their mechanisms of action, laxatives are classified into four main categories: bulk-forming laxatives, emollients/lubricants, contact/stimulant laxatives, and osmotic laxatives. Among them, osmotic and stimulant laxatives are considered the first-line medications to alleviate constipation in adults [7]. Osmotic laxatives are widely used in chronic kidney disease (CKD), but their effects may be limited, especially in HD patients. In addition, magnesium and sodium-containing osmotic laxatives may induce adverse renal and metabolic disturbances [8,9]. Therefore, contact laxatives are usually prescribed for HD patients with constipation.

Prolonged use of laxatives, however, has been found associated with an increased risk of adverse cardiovascular events and mortality in several clinical studies [10,11,12]. The underlying mechanisms remain unclear and could be multi-factorial. Earlier experimental studies indicated several possible mechanisms in which prolonged use of laxatives was linked to cardiovascular diseases (CVD) in animals. For example, the destruction of the gut mucosa barrier and flora would induce bacterial overgrowth and eventually lead to chronic inflammatory response and the development of atherosclerosis [13] or the formation of vasoconstrictor factors (serotonin and prostaglandin) [14]. In addition, diarrhea, dehydration, and electrolyte imbalances are common side effects of contact laxatives use [15], which may compromise vascular hemodynamics. Therefore, contact laxative use is not only an indicator but also increases the risk for CVD.

Arteriovenous fistula (AVF), which is created by a surgical anastomosis of the patient’s native artery and vein, is the best option for vascular access. Arteriovenous fistula (AVF) was created by a surgical anastomosis of the patient’s native artery and vein, is the best option of vascular access (VA) for maintenance HD, owing to its prolonged patency, optimal blood flow, few complications, and low maintenance costs [16,17]. However, the non-maturation rate remains high; approximately 23–50% of all AVFs fail to achieve maturation, resulting in repeated surgical or radiological interventions or eventually switching to alternative types of VA [18,19]. AVF maturation is a vascular remodeling process that normally takes 6–12 weeks after surgical anastomosis. Multiple factors that affect AVF maturation include age, gender, diabetes, obesity, vascular features, surgical technique and surgeon expertise, preoperative planning, and mapping [20]. Additionally, several medications have been mentioned to be related to AVF maturation and patency [21,22,23]. Given that AVF is part of the cardiovascular system, its maturation could be influenced by similar risk factors for CVD. As mentioned above, the use of contact laxatives has been considered a risk factor for CVD. However, very limited studies investigate the relationship between contact laxative use and the AVF maturation outcome. Therefore, this study was conducted to explore the association of contact laxative use with AVF maturation outcomes in patients undergoing HD.

## 2. Materials and Methods

### 2.1. Study Design and Data Source

We performed a multi-center retrospective cohort study using the Taipei Medical University Clinical Research Database (TMUCRD), which included all medical claims of patients who visited three affiliated hospitals, including Taipei Medical University Hospital, Wan Fang Hospital, and Shuang Ho Hospital. This study was approved by the joint institutional review board (IRB) committee of Taipei Medical University (N202105032). All data were anonymized and de-identified by scrambling the patients’ identification codes and medical facilities before acquisition for analysis.

### 2.2. Study Population

**Figure 1:**
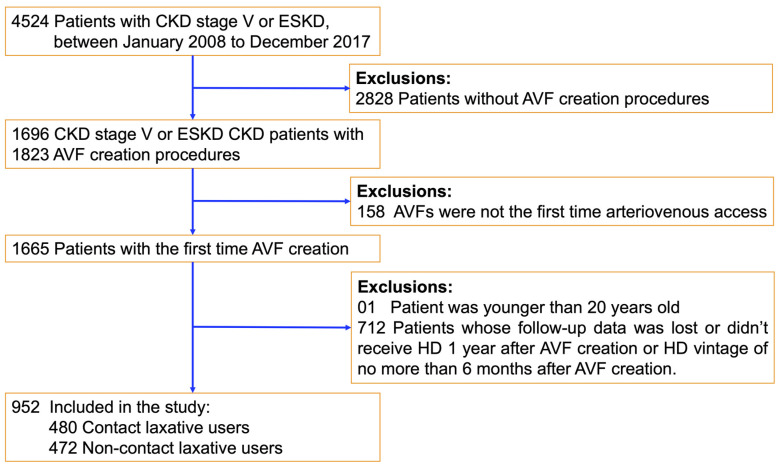
Enrollment process of the study population. Note: CKD—chronic kidney disease; ESKD—end-stage kidney disease; AVF—arteriovenous fistula; HD—hemodialysis.

We first identified patients who were diagnosed with CKD stage 5 or end-stage renal disease (ESRD) based on the International Classification of Disease, Ninth Revision [ICD-9] codes: 585.5, 585.6 and Tenth Revision [ICD-10] codes: N18.5, N18.6. From them, advanced CKD patients who received their first AVF creation (based on procedure codes under Taiwan’s National Health Insurance (NHI): F69032A, F69032B) between 1 January 2008 and 31 December 201, were included in the study. These AVFs are the initial long-term vascular access for HD patients and are most commonly a Cimino type (The preferred distal AVF), which are creased by a surgical anastomosis of the radial artery and the cephalic vein at the level of the wrist using “the end-to-side” technique [20]. Patients who were under the age of 20 years old at the time of AVF creation were excluded. Each patient was followed up at least one year after the AVF creation at the three affiliated hospitals of Taipei Medical University to monitor and confirm the outcome of AVF maturation (See Figure 1).

### 2.3. Contact Laxative Exposures

TMUCRD has recorded information on all prescribed medications from three affiliated hospitals of Taipei Medical University. Contact laxatives are identified as Anatomical Therapeutic Chemical (ATC) code A06AB; other laxatives are A06AA, A06AC, and A06AD. For each study participant, the data related to the contact laxative use were included the date of dispensing, the average daily dose (aDD), and the cumulative treatment days (cTD) within three months before and after the date of AVF creation. These medications could be used for temporary relief of acute constipation or longer duration for chronic constipation. The patients who did not use contact laxatives or use with cTD for less than seven days within the six months were defined as “non-contact laxative users”.

### 2.4. Outcome Measurement

The successful AVF maturation was determined at the time of successful use for HD, which means AVF could be provided with prescribed HD consistently with two-needle insertion for at least two-thirds of HD sessions in four consecutive weeks [24]. To achieve that, the AVFs need to mature in diameter and wall thickness to allow easy cannulation and to provide sufficient blood flow for the dialysis sessions. On the contrary, maturation failure was determined if the AVF could not be used for HD despite further interventions. After AVF creation, all patients were monitored for at least one year to document all VA events (i.e., surgical or radiological interventions, re-establishment of other types of VA) and dialysis sessions to determine the functional maturation and patency of AVFs. The patients who did not start HD one year after the date of AVF creation were excluded to eliminate those prolonged unused AVFs. In addition, the patients with dialysis vintage no more than six months after AVF creation were excluded to avoid the undetermined status of AVF maturation.

### 2.5. Measurement of Covariates

Based on NHI codes, we identified all procedures relating to dialysis treatment, including HD and peritoneal dialysis (PD), other permanent VA procedures (arteriovenous graft creation; permanent catheter insertion), and VA intervention procedures (surgical interventions and radiological interventions) (See Table S1). According to the date of AVF creation, we further determined whether the AVF creation was established before or after HD initiation, history of PD, and history of previous VA use.

We used diagnosis codes (ICD-9) from outpatient and inpatient datasets to identify major comorbidities. All diseases from the Charlson Comorbidity Index (CCI) [25] and other conditions such as hypertension, disorders of lipid metabolism, septicemia, anxiety, and depression were included in the analysis. These diseases were confirmed if at least one outpatient or inpatient visit was documented within three months before and after the AVF creation.

Other medications reported affecting AVF maturation outcome or cardiovascular system, including antiplatelets, ESAs, nitrates, beta-blockers, calcium channel blockers, angiotensin-converting enzyme (ACE) inhibitors, angiotensin II receptor blockers (ARBs), statins, and loop diuretics were examined [21,22,23]. The prescription claims of patients’ medications were also tracked and collected their aDD and cTD for up to 3 months before and after the AVF creation (See Table S2). To ensure the study patients were treated at the hospitals of Taipei Medical University, we excluded the patients who were not followed up from three months before to one year after the AVF creation. These medications have been used in HD patients for the long term, and we considered the patients who did not use these medications or used with cTD for less than thirty days within the six months were defined as “non-drug users”.

We also retrieved some routine blood tests from laboratory datasets, such as hemoglobin (Hgb), white blood cells (WBC), platelets (PLT), blood urea nitrogen (BUN), creatinine, calcium (Ca), phosphorus (P), sodium (Na), and potassium (K). The value of each blood test was calculated by its average value within three months before and after the AVF creation.

### 2.6. Statistical Analysis

Baseline demographics, history of dialysis and VA procedures, comorbidity, and medications characteristics were compared between the two groups, contact laxative users versus non-users, using chi-squared statistics. Continuous variables such as age, CCI score, and blood tests were compared between the two groups using *t*-test statistics. To inspect the relationship between contact-laxative use and the risk of AVF maturation failure, we employed a conditional logistic model to calculate the odds ratios (ORs) and 95% confidence intervals (CIs). The adjusted ORs (aOR) were estimated using multiple logistic regression models; the ORs were adjusted for sex, age, history of dialysis and vascular access, age-unadjusted CCI score, other medications, and blood tests.

Furthermore, we stratified contact laxative users into different subgroups based on aDD and cTD. For aDD, we categorized patients into non-users, low dose with aDD < 15 mg, and high dose with aDD ≥ 15 mg. Meanwhile, for cTD, patients were put into non-users, short term with cTD < 60 days or 60 days ≤ cTD < 90 days, and long term with cTD ≥ 90 days. These well-defined groups allowed us to identify the relationship between the risk of AVF maturation failure and the changes in dose and duration of contact laxative administration using multiple logistic regression estimation. The data were analyzed using IBM^®^SPSS^®^Statistics (IBM, Armonk, NY, USA) (version 26.0 for Windows). The results were considered statistically significant with a two-tailed *p* < 0.05.

## 3. Results

### 3.1. Baseline Characteristics

We screened 4524 advanced CKD patients between 1 January 2008 and 31 December 2017. After excluding those who did not meet the study criteria, 952 advanced CKD patients with their first AVF creation documented were included in this study (see Figure 1). Of them, 480 contact laxative users and 472 non-users were identified.

Demographic characteristics, history of dialysis and VA procedures, comorbidities, CCI score, medications, and routine blood tests of the study population are shown in Table 1. The mean age (standard deviation, SD) was 63.6 (13.42) years; 38.2% of patients were female, and 60.7% of patients were diabetic. Compared with non-users, contact-laxative users were older and more likely to be dialysis patients. They had a significantly higher prevalence of comorbidities, including septicemia, diabetes, hypertension, ischemic heart disease, congestive heart failure, cerebral vascular disease, peripheral vascular disease, chronic pulmonary disease, peptic ulcer disease, anxiety, and depression. They also had higher CCI scores than those non-users (age-unadjusted CCI scores (SD), 3.63 (1.44) for contact-laxative users vs. 3.10 (1.17) for non-users, *p* < 0.0001). Except for loop diuretics which were more commonly used in contact laxative users than non-users, the use of other medications showed no significant difference between the two groups. Regarding blood tests, contact laxative users had lower levels of serum creatinine, phosphorus (P), and potassium (K) than non-users.

### 3.2. AVF Maturation Outcome in the Contact Laxative Users and Non-Users

After adjusting for potential confounders through multiple logistic regression, the association between contact laxative use and an increased risk of AVF maturation failure was found (aOR, 1.63; 95% CI 1.17–2.26; *p* = 0.004).

**Figure 2:**
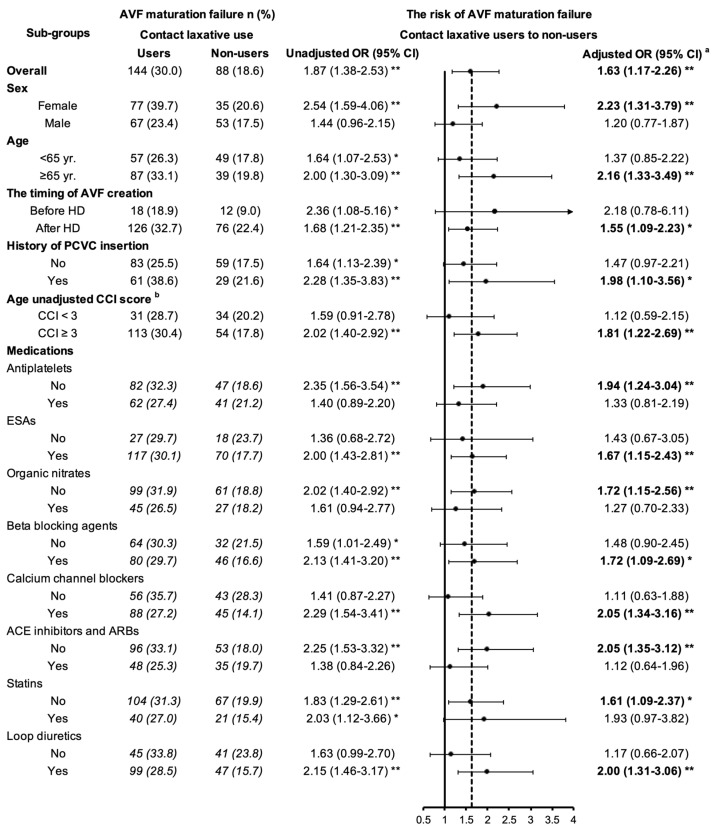
Laxatives use and their association with AVF maturation failure by different covariates. Note: AVF—arteriovenous fistula; yr.—year; HD—hemodialysis; PD—peripheral dialysis; CCI—Charlson Comorbidity Index; ESAs—erythropoietin stimulating agents; ACE inhibitors—angiotensin-converting enzyme inhibitors; ARBs—angiotensin receptor blockers. ^a^ Adjusted OR (adjusted odds ratio) was estimated using multiple logistic regression model and adjusted for covariate factors, including age, gender, history of dialysis and vascular access, Charlson Comorbidity Index score, laboratory examinations, and medications listed in Table 1. ^b^ Charlson score represents the degree of health; a high score indicates a worse health condition. * *p* < 0.05; ** *p* < 0.01.

Multivariable stratified analyses, including patient demographics, history of dialysis and vascular access, comorbidities, and medications, are shown in Figure 2. Contact laxative use was found to be associated with an increased risk of AVF maturation failure only in females (aOR, 2.23; 95% CI, 1.31–3.79; *p* = 0.003); those older than 65 years (aOR, 2.16; 95% CI, 1.33–3.49; *p* = 0.002); those who created AVF after HD initiation (aOR, 1.55; 95% CI, 1.09–2.23; *p* = 0.015); those with history of permanent central venous catheter (PCVC) (aOR, 1.98; 95% CI, 1.10–3.56; *p* = 0.023); and those with age-unadjusted CCI score ≥3 (aOR, 1.81; 95% CI, 1.22–2.69; *p* = 0.003). Notably, it was demonstrated that an increased risk of AVF maturation failure only when contact laxatives used in combination with erythropoietin stimulate agents (aOR, 1.67; 95% CI, 1.15–2.43; *p* = 0.007), beta-blocking agents (aOR, 1.72; 95% CI, 1.09–2.69; *p* = 0.019), calcium channel blockers (aOR, 2.05; 95% CI, 1.34–3.16; *p* = 0.001), or loop diuretics (aOR, 2.00; 95% CI, 1.31–3.06; *p* = 0.001). In contrast, there was no significant difference in the risk of AVF maturation failure when contact laxatives were used with antiplatelets, nitrates, ACE inhibitors and ARBs, or statins.

Finally, analyses based on aDD and cTD within three months before and after the AVF creation revealed the dose- and duration-response effects, respectively. When taking into consideration of aDD and cTD together, the aORs for AVF maturation failure increased respectively in four groups from “low dose + short term”, “ low dose + long term”, “ high dose + short term”, and “high dose + long term”, compared to the non-users (See Table 2), but only contact laxative users with high doses and long duration had a significantly increased risk of AVF maturation failure (aOR, 2.38; 95% CI, 1.50–3.79; *p* < 0.0001), compared to the non-users.

## 4. Discussion

In this multi-center cohort study, we demonstrated the association of contact laxative use with AVF maturation outcomes in advanced CKD patients who received their first AVF creation. Contact laxative use was significantly associated with an increased risk of AVF maturation failure. Subgroup analysis further identified differential risks of contact laxative use under different patient characteristics or clinical settings. Finally, the use of contact laxatives at a high dose (aDD ≥ 15 mg) and with a long duration (cTD ≥ 90 days) had the highest risk of AVF maturation failure compared to non-users as well as those receiving a low dose or short period.

Several potential mechanisms might explain the association between contact laxative use and AVF maturation outcomes. First, the chance of intestinal secretions and motility could be increased due to contact laxatives acting as a stimulus of myenteric and Auerbach plexuses. In addition, low doses of stimulative laxatives inhibit water and sodium absorption, but high doses increase sodium release into the colonic lumen, followed by water [26]. Therefore, high doses and/or prolonged use of contact laxatives can cause diarrhea. The association between contact laxative used and an increased risk of diarrhea was shown in a recent meta-analysis (risk ratio, 13.75; 95% CI, 2.82–67.14) compared with the placebo [27]. Diarrhea is often accompanied by dehydration and electrolyte loss, which may alter hemodynamics (hypotension) and potentially lead to AVF dysfunction/occlusion [28,29]. Second, contact laxatives may induce intestinal mucosal barrier injury and gut dysbiosis. In 1984, a study by Dufour et al., which used sennoside A (a form of contact laxatives) for over 16 weeks on normal mice, showed potential damage to the intestinal mucosa and destruction of the intestinal wall structure due to the drug [30]. According to another research, intestinal epithelial barrier disruption, bacterial overgrowth, and chronic inflammation in mice were also found to be associated with the use of Sennoside A in a dose-dependent manner [31]. The casual relationship between gut microbiota and the development of atherosclerosis has been highlighted recently [32,33]. Atherosclerosis may cause luminal narrowing and loss of vascular elasticity (increase in arterial stiffness), which in turn inhibit the outward remodeling process during AVF maturation [34,35]. Third, some contact laxatives could induce the formation of serotonin [14], which then causes vasoconstriction and increase smooth muscle cell aggregation [36,37]. Vasoconstriction might decrease the blood flow in AVF, and smooth muscle cell proliferation and migration would promote intimal hyperplasia [38]; both are primary reasons for AVF failure [39].

Interestingly, our results indicated that using contact laxatives combined with ESAs, beta-blocking agents, calcium channel blockers, or loop diuretics might augment the risk of AVF maturation failure. Indeed, a previous experimental study has shown that ESAs could stimulate vascular smooth muscle cell proliferation and induce neointimal hyperplasia [40], and become a risk factor for AVF maturation failure, especially at high doses or long-term treatment duration [23,41]. Meanwhile, constipation is considered a side effect of calcium channel blockers [42]. When used in conjunction with contact laxatives, diuretics could worsen electrolyte disturbances and dehydration [11]. All these factors might synergistically compromise the maturation of AVF. Therefore, caution should be taken when these drugs are co-administered with contact laxatives.

There are several limitations to the present study. First, the direct causal relationship between contact laxative use and AVF maturation failure cannot be inferred based on the nature of the observational study. Second, patients who had received AVF creation at three affiliated hospitals of Taipei Medical University but later on continued HD at other hospitals were excluded from the study. That could generate a selection bias. Moreover, it should be noted that several contact laxatives are available over-the-counter from pharmacies and supermarkets. Thus, the use of laxatives might be underestimated. However, patients with advanced CKD in Taiwan (from CKD stage 3b to stage 5) are enrolled in the nationwide pre-ESRD program, which provides comprehensive care and education, including medication use, under the guidance of a physician. Additionally, these laxatives are fully covered by Taiwan National Health Insurance. Therefore, most laxatives used in patients undergoing HD should be documented in this database. Third, there was a lack of information related to the skill and experience of surgeons in this study. However, the three university-affiliated hospitals are expected to have a similar standard of care and qualification for surgeons. Finally, constipation itself could result in gut dysbiosis and has been linked to increased adverse cardiovascular events [10,33]. Hence, AVF maturation failure could be attributable to both constipation and contact laxative use overlappingly. Future studies should be well designed to address their independent roles in this regard.

## 5. Conclusions

In conclusion, we observed a dose- and the duration-dependent association between contact laxative use and increased risk of AVF maturation failure in patients undergoing HD. Although several experimental evidence might explain the mechanisms underneath these observations, further studies are needed to delineate the exact causal relationship between contact laxative use and AVF maturation failure.

## Figures and Tables

**Table 1:** Patient characteristics of the study population.

Variables	Overall	Contact Laxative Group (n = 480)	Non-Contact Laxative Group (n = 472)	*p*-Value ^a^
**Age, n (%)**				<0.0001
Age < 65 yr.	492 (51.7)	217 (45.2)	275 (58.3)	
Age ≥ 65 yr.	460 (48.3)	263 (54.8)	197 (41.7)	
Mean (SD)	63.6 (13.4)	66.4 (12.9)	60.8 (13.4)	<0.0001
**Sex, n (%)**				0.163
Female	364 (38.2)	194 (40.4)	170 (36.0)	
Male	588 (61.8)	286 (59.6)	302 (64.0)	
**The timing of AVF creation, n (%)**				0.002
Before HD	228 (23.9)	95 (19.8)	133 (28.2)	
After HD	724 (76.1)	385 (80.2)	339 (71.8)	
**History of PD, n (%)**				0.082
No	897 (94.2)	446 (92.9)	451 (95.6)	
Yes	55 (5.8)	34 (7.1)	21 (4.4)	
**History of the long-term catheter, n (%)**				0.130
No	660 (69.3)	322(67.1)	338 (71.6)	
Yes	292 (30.7)	158 (32.9)	134 (28.4)	
**Age-unadjusted CCI scores, n (%) ^b^**				<0.0001
CCI < 3	276 (29.0)	108 (22.5)	168 (35.6)	
CCI ≥ 3	676 (71.0)	372 (77.5)	304 (64.4)	
Mean (SD)	3.37 (1.34)	3.63 (1.44)	3.10 (1.17)	<0.0001
**Comorbidities, n (%)**				
Septicemia	83 (8.7)	56 (11.7)	27 (5.7)	0.001
Malignant neoplasms	65 (6.8)	39 (8.1)	26 (5.5)	0.110
Diabetes mellitus	578 (60.7)	325 (67.7)	253 (53.6)	<0.0001
Disorders of lipid metabolism	280 (29.4)	150 (31.3)	130 (27.5)	0.209
Hypertension	767 (80.6)	401 (83.5)	366 (77.5)	0.019
Ischemic heart disease	299 (31.4)	166 (34.6)	133 (28.2)	0.033
Cardiac dysrhythmias	111 (11.7)	59 (12.3)	52 (11.0)	0.540
Congestive heart failure	326 (34.2)	197 (41.0)	129 (27.3)	<0.0001
Cerebral vascular disease	114 (12.0)	75 (15.6)	39 (8.3)	<0.0001
Peripheral vascular disease	42 (4.4)	28 (5.8)	14 (3.0)	0.031
Chronic pulmonary disease	91 (9.6)	55 (11.5)	36 (7.6)	0.044
Liver diseases	54 (5.7)	29 (6.0)	25 (5.3)	0.619
Peptic ulcer disease	160 (16.8)	96 (20.0)	64 (13.6)	0.008
Anxiety and depression	52 (5.5)	35 (7.3)	17 (3.6)	0.012
**Laboratory data, Mean (SD)**				
HGB (g/dL)	9.48 (1.09)	9.46 (1.07)	9.50 (1.11)	0.561
WBC (10^3^/uL)	7.43 (2.35)	7.56 (2.31)	7.29 (2.38)	0.077
PLT (10^3^/uL)	187.8 (63.1)	187.4 (62.8)	188.1 (63.5)	0.875
BUN (mg/dL)	79.0 (26.5)	78.1 (26.22)	79.9 (26.7)	0.318
Creatinine (mg/dL)	9.10 (2.96)	8.62 (2.76)	9.59 (3.08)	<0.0001
Ca (mg/dL)	8.33 (0.77)	8.36 (0.79)	8.30 (0.75)	0.169
P (mg/dL)	5.48 (1.47)	5.32 (1.43)	5.64 (1.50)	0.001
Na (mmol/L)	136.3 (3.12)	136.2 (3.22)	136.4 (3.01)	0.257
K (mmol/L)	4.47 (0.62)	4.38 (0.62)	4.55 (0.60)	<0.0001
**Medications, n (%)**				
Antiplatelets	419 (44.0)	226 (47.1)	193 (40.9)	0.054
ESAs	783 (82.5)	389 (81.0)	396 (83.9)	0.247
Organic nitrates	318 (33.4)	170 (35.4)	148 (31.4)	0.184
Beta blocking agents	546 (57.4)	269 (56.0)	277 (58.7)	0.409
Calcium channel blockers	643 (67.5)	323 (67.3)	320 (67.3)	0.868
ACE inhibitors and ARBs	368 (38.7)	190 (39.6)	178 (37.7)	0.553
Statins	284 (29.8)	148 (30.8)	136 (28.8)	0.496
Loop diuretics	647 (68.0)	347 (72.3)	300 (63.6)	0.004

Note: SD—standard deviation; yr.—years; AVF—arteriovenous fistula; HD—hemodialysis; PD—peritoneal dialysis; CCI—Charlson Comorbidities Index; ESAs—erythropoiesis-stimulating agents; ACE inhibitors—angiotensin-converting enzyme inhibitors; ARBs—angiotensin receptor blockers. ^a^ *p*-value was calculated using Student *t*-test with continuous variables and chi-square or Fisher exact test with categorical variables. ^b^ Charlson score represents the degree of health; a high score indicates a worse health condition.

**Table 2:** The risk of AVF maturation failure is stratified by the average daily dose, the treatment days, and the different doses and treatment duration of contact laxatives.

	AVF Maturation Failure, n (%)	AVF Maturation Success, n (%)	Adjusted OR (95% CI) ^a^	*p*-Value
Contact laxatives (aDD, mg)				
Non-users (ref.)	88 (18.6)	384 (81.4)	1.00	-
aDD < 15	60 (26.0)	171 (74.0)	1.36 (0.91–2.03)	0.135
aDD ≥ 15	84 (33.7)	165 (66.3)	1.91 (1.31–2.80)	0.001
Contact laxatives (cTD, days)				
Non-users (ref.)	88 (18.6)	384 (81.4)	1.00	-
cTD < 60	31 (23.1)	103 (76.9)	1.13 (0.69–1.86)	0.615
60 ≤ cTD < 90	33 (30.6)	75 (69.4)	1.80 (1.09–2.97)	0.021
cTD ≥ 90	80 (33.6)	158 (66.4)	1.89 (1.28–2.80)	0.001
Contact laxatives (aDD, mg; and cTD, days)				
Non-users (ref.)	88 (18.6)	384 (81.4)	1.00	
aDD < 15 and cTD < 90	29 (23.8)	93 (76.2)	1.30 (0.79–2.15)	0.297
aDD < 15 and cTD ≥ 90	31 (28.4)	78 (71.6)	1.42 (0.85–2.39)	0.182
aDD ≥ 15 and cTD < 90	35 (29.2)	85 (70.8)	1.51 (0.92–2.46)	0.101
aDD ≥ 15 and cTD ≥ 90	49 (38.0)	80 (62.0)	2.38 (1.50–3.79)	<0.0001

Note: AVF—arteriovenous fistula; OR—odds ratio; CI—confidence intervals; aDD—average daily dose; cTD—cumulative treatment days; ref. —reference. ^a^ Adjusted OR (adjusted odds ratio) was estimated using multiple logistic regression model and adjusted for covariate factors, including age, gender, history of dialysis and vascular access, Charlson Comorbidity Index score, laboratory examinations, and medications listed in Table 1.

## Data Availability

The data presented in this study are available on request from the corresponding author. The data are not publicly available due to privacy restrictions.

## Supplementary Materials

The following supporting information can be downloaded at: https://www.mdpi.com/article/10.3390/ijerph19116842/s1, Table S1: Dialysis and vascular access procedures related codes in this study; Table S2: List of medications used in this study.
