# Supplementary material for: Contact Laxative Use and the Risk of Arteriovenous Fistula Maturation Failure in Patients Undergoing Hemodialysis: A Multi-Center Cohort Study
Source: Int J Environ Res Public Health. 2022 Jun 3;19(11):6842. doi: 10.3390/ijerph19116842 (PMC9180587; doi:10.3390/ijerph19116842)
Supplement: Supplementary file 1 [file ijerph-19-06842-s001.zip › ijerph-1717474-supplementary.pdf]

Title

Contact Laxative Use and the Risk of Arteriovenous Fistula Maturation Failure in Hemodialysis Patients: A Multi-Center Cohort Study

Trung Hoang Anh, Phung Anh Nguyen, Anh Duong, I-Jen Chiu, Chu-Lin Chou, Tzu-Hao Chang, Chih-Wei Huang, Mai-Szu Wu, Chia-Te Liao, Yung-Ho Hsu

Title..........1
Table S1: Dialysis and vascular access procedures related codes in this study..........2
Table S2: List of medications use in this study..........3

Table S1: Dialysis and vascular access procedures related codes in this study.

Procedures	NHI code
**Vascular access procedures for hemodialysis**	
AVF creation	69032C
AVG creation	69034C
PCVC insertion	69039B
	69039B
	47061B
	47059B
PCVC removal	62009C
TCVC insertion	69006C
	47065B
	47084B
Surgical intervention	69038C
	69003B
	69001B
	69002B
Radiological intervention	58004C
	58006C
	58005C
	33074B
	33115B
**Dialysis procedures**	
Hemodialysis	58001C
	58019C
	58020C
	58021C
	58022C
	58023C
	58024C
	58025C
	58029C
Peritoneal dialysis	58002C
	58009B
	58010B
	58011C
	58017B
	58017C
	58012B

Note: NHI, National health insurance; AVF, Arteriovenous fistula; AVG, Arteriovenous graft; PCVC, Permanent central venous catheter; TCVC, Temporary central venous catheter.

Table S2: List of medications use in this study.

ATC code	ATC name
**A06AA**	Softeners, emollients
**A06AB**	Contact laxatives
**A06AC**	Bulk-forming laxatives
**A06AD**	Osmotically acting laxatives
**B01AC**	Antiplatelets
**B03XA01, B03XA02, B03XA03**	Erythropoietin stimulating agents
**C01DA**	Nitrates
**C07**	Beta-blockers
**C08**	Calcium channel blockers
**C09A, C09C**	ACE inhibitors, angiotensin II antagonists
**C10AA**	Statins
**C03CA**	Loop diuretics

Note: ATC, Anatomical Therapeutic Chemical
